# Street‐vended grilled beef sausages as potential vehicles of bacterial and fungal pathogens: An exploratory survey in Ho, the capital city of the Volta Region of Ghana

**DOI:** 10.1002/fsn3.3625

**Published:** 2023-08-31

**Authors:** Banabas Nkekesi, Priscilla Amenya, George Aboagye, Nii Korley Kortei

**Affiliations:** ^1^ Department of Nutrition and Dietetics, School of Allied Health Sciences University of Health and Allied Sciences Ho Volta Region Ghana

**Keywords:** *Aspergillus niger*, grilled beef sausages, pathogens, ready‐to‐eat, *Salmonella* spp., street‐vended food

## Abstract

Grilled beef sausage is a popular street delicacy in many countries, and Ghana is no exception. This study assessed street‐vended grilled beef sausages as a potential vehicle of microorganisms that present food safety risks to the general public in Ho City. Twenty grilled beef sausages were obtained from various vended locations within Ho municipality by convenient sampling and were analyzed by standard microbiological protocols for food safety followed by statistical analysis with a test of significant difference at *p* < .05. Total aerobic bacteria count of the potential foodborne pathogens ranged from 2.75 × 10^4^ to 1.85 × 10^7^ CFU/g. The microbial species identified included *Staphylococcus aureus* with a load from 6.15 × 10^2^ to 1.67 × 10^5^ CFU/g, *Escherichia coli* from 4.2 × 10^2^ to 3.9 × 10^4^ CFU/g, *Bacillus cereus* from 3.05 × 10^2^ to 7.1 × 10^4^ CFU/g, and *Salmonella* spp. from 2.8 × 10^2^ to 5.5 × 10^4^ CFU/g. Total fungal counts also ranged from 0.0 to 9.83 × 10^3^ CFU/g, and the species identified included *Aspergillus* spp. and *Rhizopus* spp. all of which were within the acceptable limits of the International Commission for Microbiological Specification of Foods. However, for total viable bacteria, 75% of the samples were above the acceptable limits in the guidelines by the Ghana Standards Authority, indicating that the consumption of grilled beef sausages poses serious food safety and hygiene risks to consumers. Hygienic processing of the sausages under sanitary environments, proper handling and preservation procedures, and periodic follow‐up visits to the vended areas should be employed to reduce the risk of occurrence of potential pathogens in the products.

## Graphical abstract

Grilled beef sausages sold by kebab vendors in Ho City were analyzed by food safety standard protocols to determine the load, type, and frequency of occurrence of both bacterial and fungal species. Findings provided grounds that the sausages could serve as potential vehicles of foodborne illness to the people of Ho City.

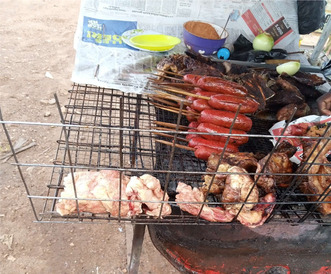

## 1 BACKGROUND

Meat and meat products are considered significant boosters for growth and development due to their nutritional properties that provide significant levels of proteins, fatty acids, vitamins, minerals, and other bioactive compounds (Decker & Park, 2010). Sausage is considered a type of meat product that is consumed in different countries across the world and it is a mass‐consumer fast food. Beef sausage is prepared from minced beef with salt, spices, fillers, and permitted binders. Beef sausage as a source of animal protein is greatly consumed in Ghana and dietitians or nutritionists recommend it as a major source of protein for growing children, immunocompromised patients, expectant mothers, and the aged.

Meat processing converts meat by employing physical and biochemical technologies that add value to the product (Teye, 2010), leading to the preservation or extension of shelf life, and improvement of tenderness and flavor of meat and its products (Heinz & Hautzinger, 2007). Meat and meat products, in general, have been implicated as vehicles for the spread of food‐borne pathogens (Altajori & Elshrek, 2014). They provide adequate nutrients that encourage the growth of microorganisms, rendering meat perishable. Due to its nature, sausage presents an increased surface area which can easily get contaminated when not properly stored. Many interrelated factors such as holding temperatures, atmospheric oxygen, endogenous enzymes, moisture, light, and most importantly microorganisms influence the shelf life and freshness of meat (Eie et al., 2007; Zhou et al., 2010). The microorganisms that had been isolated from different sausage products and processing lines include *Staphylococcus aureus*, *Bacillus cereus*, *Escherichia coli*, *Salmonella*, *Listeria monocytogenes*, yeasts, and molds (Afshin et al., 2011; Altajori & Elshrek, 2014; Güngör & Gökoğlu, 2010).

The consumption of grilled beef sausage has become very popular in Ghana and beyond. Beef sausage is grilled on a glowing charcoal fire, and is stacked on wood sticks and spiced with vegetable oil to improve its flavor. It is usually served with onions and powdered pepper. The ready‐to‐eat beef sausages are often prepared and sold along the streets, in clubhouses, restaurants, and hotels under unhygienic conditions, and are normally packaged in nonsterile newspapers. Street foods play a key socioeconomic role in meeting the food and nutritional needs of city consumers at affordable prices for the lower and middle‐income classes and are valued for their unique flavors and convenience (Khairuzzaman et al., 2014). However, serious concerns exist about the handling and safety of street‐vended foods in developing countries (Imathiu, 2017). According to Rane (2011), high levels of pathogenic bacteria capable of causing food poisoning have been quantified in these foods and have been declared unacceptable for consumption. It is, therefore, imperative that attempts are made to reduce the high levels of bacterial contamination to acceptable levels.

**FIGURE 1:**
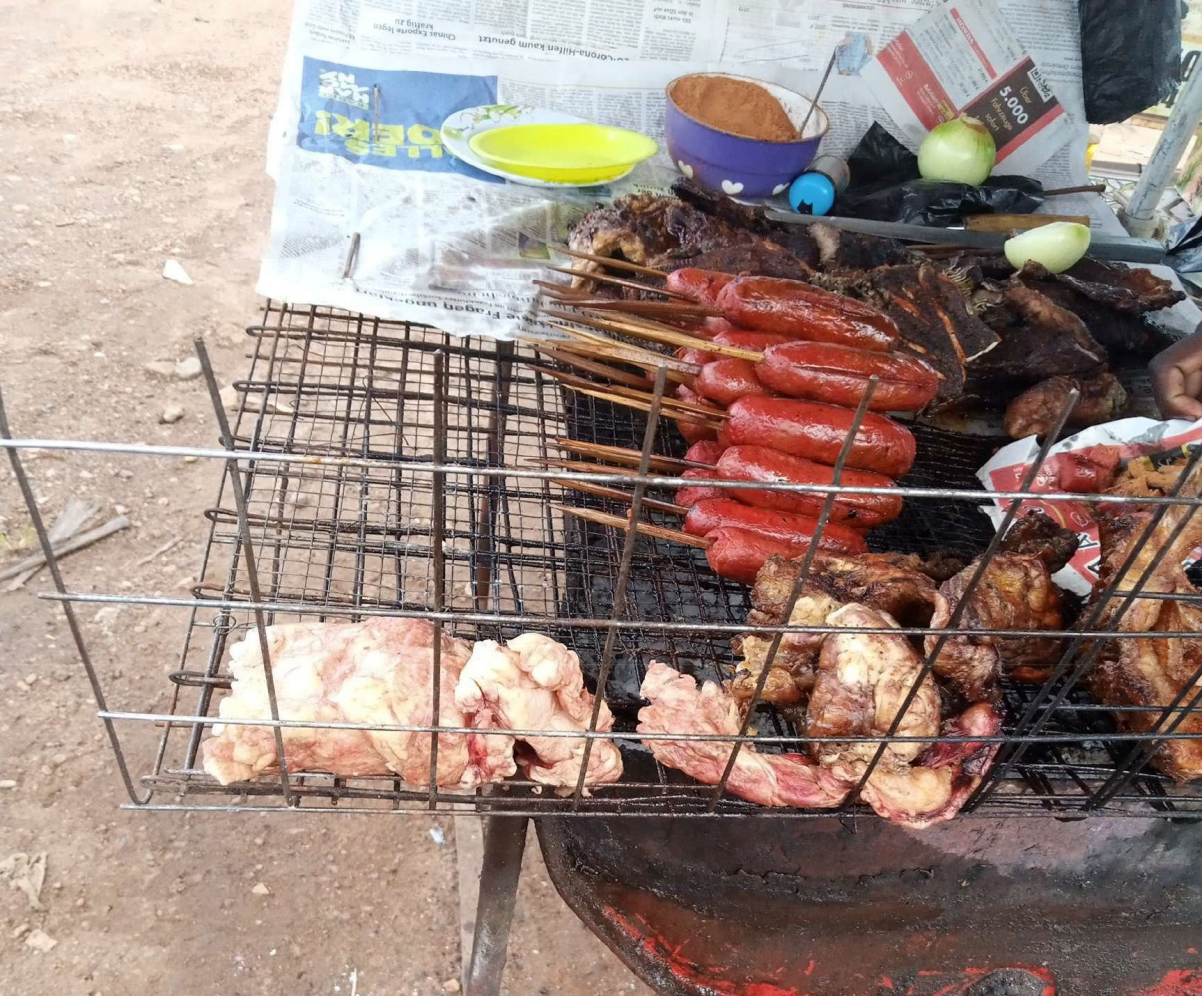
A showcase of grilled beef sausages sold at a vending area in Ho municipality.

In Ghana, most street vendors operate without regulation or monitoring by the responsible authorities on the foods they sell. Kebab sellers often sell grilled sausages in an open space or along the streets which are exposed to the environment. Most of these vendors typically operate in the evening until night or daybreak and do not adhere to recommended meat safety practices (Adzitey et al., 2020). According to Atter et al. (2020), most processors purchase fresh sausages from the cold store during the period it is kept in a freezer, but the major issue is the applicable storage practices before grilling. Although grilling of beef sausages may kill all bacteria, yeast, and molds, rendering them harmless due to the heat applied during preparation (Figure 1), the operations of the meat vendors in an open space make it possible for contamination by physical, chemical, or biological hazards from the environment (Adzitey, 2016).

Among the biological hazards are *Salmonella*, *Escherichia coli*, *Bacillus cereus*, *Staphylococcus aureus*, yeast, and molds. The consumption of grilled beef sausages, therefore, presents various risk levels to consumers based on the frequency of intake and hygienic practices executed by the vendors. Therefore, this study investigated the microbiological quality of street‐vended grilled beef sausages, the first of its kind in the capital city of the Volta Region of Ghana to ascertain the risks in handling preparations and consumption to provide safety recommendations to consumers.

## 2 METHODS

### 2.1 Sampling

Samples were collected randomly from 20 selected vending areas in the Ho municipality. The main reason for the selection of these areas was based on population density as well as patronage for the grilled beef sausage. The specific sampling point of the selected vending areas was recorded as follows: Dave vending area (Sample A), Trafalgar vending area (Sample B), Melcom vending area (Sample C), Civic Center vending area (Sample D), Dome vending area (Sample E), Market vending area (Sample F), Fiave vending area (Sample G), Zongo vending area (Sample H), Barracks vending area (Sample I), Guinness vending area (Sample J), Ahoe vending area (Sample K), Poly vending area (Sample L), Lokoe vending area (Sample M), SSNIT Flat vending area (Sample N), Bankoe vending area (Sample O), Mawuli Estate vending area (Sample P), Mirage vending area (Sample Q), Alaye vending area (Sample R), Ola vending area (Sample S), and Housing vending area (Sample T). A total of 40 grilled beef sausage vendors were identified along the streets of Ho municipality. Samples of grilled beef sausage that are vended along the streets of Ho municipality were included in the study. This study was conducted using experimental design, that is, the data were based on direct observation.

### 2.2 Sample size determination

A convenience sampling technique, Hotjar sample size calculator, and Cochran's formula for smaller populations were employed to obtain 20 grilled beef sausages from kebab vendors located across the entire Ho municipality.

All samples were kept on ice immediately after being purchased and were transported to the microbiology laboratory of the University of Allied Health Sciences (UHAS) for microbiological analysis.

### 2.3 Microbiological analysis

Bacterial species were enumerated and identified by the procedures described by Aboagye et al. (2020) and Dortey et al. (2020) whilst fungal species were determined as described by Kortei et al. (2018) and Moss (1998).

### 2.4 Sample preparation

Ten grams of each sausage sample was homogenized in 90 mL of sterile distilled water. Six serial dilutions were prepared, and aliquots of 1 mL of the dilutions were plated on the respective selective media, and incubated at 37°C for up to 48 h and 7 days for bacterial and fungal species, respectively.

The selective media used were products of OXOID and BIOMARK laboratories. They included nutrient agar (Biomark Laboratories) for the isolation of total aerobic bacteria count, MacConkey agar (Biomark Laboratories) for the isolation of *Escherichia coli*, *Salmonella‐Shigella* Agar (OXOID CM0099) for the isolation of *Salmonella* spp., Mannitol salt agar (OXOID CM0085) for the isolation of *Staphylococcus aureus*, *Bacillus cereus* selective agar base (OXOID CM0617) for the isolation of *Bacillus cereus*, oxytetracycline–glucose–yeast extract agar (OXOID CM0545) for the isolation of yeast, and Sabouraud dextrose agar (OXOID CM0041) for the isolation of molds. All media were prepared using the manufacturer's instructions.

### 2.5 Identification of bacterial and fungal species

Bacterial colonies that appeared on the media surface showed distinctive morphologies. The variation in bacterial colony morphology was observed both visually and with the aid of a light microscope (Olympus) at x1000 magnification. The morphological variation included shape, size, color, and surface elevation on the sensitive and specific media used, whilst Standard Gram stain technique was employed for additional elaboration of the species isolated from the sausages.

Fungal species were identified using lactophenol cotton blue by employing morphological characterization including texture and color of colonies on the sensitive and specific media used. Microscopic examination was also carried out at x1000 magnification (Olympus). Slides of fungal cultures were prepared by gently lifting the mycelial mat with a sterile inoculation pin into a drop of lactophenol cotton blue, and mixed uniformly using sterile loop and the suspension laid with a coverslip followed by the microscopic examination. The morphological features of the isolated fungi were observed and identified as described by Moss (1998) in Table 1 below.

**TABLE 1:** Cultural and morphological characteristics of the fungi identified.

Fungal species	Cultural characteristics	Morphological characteristics
*Aspergillus* spp.	Yellow to green, and black colonies with distinct margin	Conidiophores arise from a foot cell. Club‐shaped vesicles at top of the conidiophores. Conidia are found in chains
*Fusarium* spp.	White‐pink sparse aerial mycelia becoming felty	Macroconidia sparse, borne on phialides on branched conidiophores (Septate banana shaped).
*Penicillium* spp.	Fast‐growing colonies in green color with dense felt conidiophore	Branched conidiophores with chains of conidia look like a brush
*Mucor* spp.	Large white colonies turn into black later	Erect sporangiophores are formed. Sporangiophore swells at the tip to form sporangia which are globular shaped. Columella is present
*Rhodotorula* spp.	Soft, smooth, moist, and mucoid	Round or oval‐shaped budding cell
*Rhizopus* spp.	White cottony mycelia, with black dots, cover the entire plate	Sporangiospores are produced inside a spherical sporangium. The columella is present on the top of the sporangiophore. Root‐like rhizoids are found

*Note*: Sources: da Cunha et al. (2013), Madrid et al. (2014).

The percentage occurrence of both bacterial and fungal species was calculated using the following formula:

$$\text{Percentage}\;\left(\%\right)\;\text{occurrence of bacteria}/\text{fungi}=\frac{\text{Number of bacteria}/\text{fungi}}{\text{Total number of bacteria}/\text{fungi isolated}}\times 100$$

### 2.6 Data analysis

The mean bacteria counts were determined along with the fungal counts as described by Kortei et al. (2018) and Odamtten et al. (2018). The data were subjected to one‐way ANOVA and values were considered for statistical significance at *p* < .05 using Statistical Package for Social Sciences (IBM SPSS) version 26. The guidelines of the Ghana Standard Authority (2019) and the International Commission for Microbiological Specification of Food (ICMSF, 1998) were used to interpret the microbiological quality of the street‐vended grilled beef sausage.

## 3 RESULTS

### 3.1 Bacterial load in the grilled beef sausages

The results obtained from the microbial analysis are presented in Table 2 below.

**TABLE 2:** Mean microbial load of grilled beef sausage samples.

Vending area	Sample	TABC	TSC	TEC	TBC	TSSC
Dave	A	3.58 × 10^6^	6.6 × 10^3^	5.2 × 10^3^	7.1 × 10^4^_a_	6.55 × 10^3^
Trafalgar	B	6.0 × 10^4^	7.25 × 10^3^	4.1 × 10^3^	3.05 × 10^2^_b_	5.2 × 10^3^
Melcom	C	3.55 × 10^4^	8.95 × 10^3^	5.75 × 10^3^	4.7 × 10^2^	NOG
Civic Center	D	8.42 × 10^4^	6.7 × 10^3^	2.62 × 10^4^	5.1 × 10^2^	5.8 × 10^2^
Dome	E	4.21 × 10^5^	4.34 × 10^4^	8.7 × 10^2^	NOG	3.05 × 10^3^
Market	F	5.33 × 10^5^	2.12 × 10^4^	2.31 × 10^4^	NOG	4.75 × 10^3^
Fiave	G	2.29 × 10^5^	2.01 × 10^4^	3.55 × 10^3^	3.6 × 10^3^	NOG
Zongo	H	1.85 × 10^7^_a_	1.67 × 10^5^_a_	1.1 × 10^4^	3.15 × 10^4^	5.5 × 10^4^_a_
Barracks	I	6.33 × 10^6^	4.12 × 10^4^	9.15 × 10^3^	4.7 × 10^3^	4.71 × 10^3^
Guiness	J	4.38 × 10^6^	8.1 × 10^3^	3.8 × 10^4^	4.95 × 10^3^	NOG
Ahoe	K	5.33 × 10^5^	7.6 × 10^3^	4.5 × 10^3^	4.75 × 10^3^	NOG
Poly	L	5.12 × 10^5^	6.35 × 10^4^	3.9 × 10^4^_a_	4.2 × 10^3^	6.55 × 10^3^
Lokoe	M	8.7 × 10^5^	8.2 × 10^3^	6.85 × 10^3^	4.05 × 10^3^	2.8 × 10^2^_b_
SSNIT Flat	N	9.0 × 10^5^	7.75 × 10^4^	3.55 × 10^3^	NOG	7.7 × 10^2^
Bankoe	O	7.25 × 10^4^	6.55 × 10^3^	4.2 × 10^2^_b_	9.6 × 10^2^	3.25 × 10^3^
Mawuli Estate	P	2.75 × 10^4^_b_	6.15 × 10^2^_b_	NOG	NOG	NOG
Mirage	Q	6.85 × 10^6^	6.75 × 10^4^	NOG	4.0 × 10^3^	NOG
Alaye	R	6.9 × 10^6^	3.7 × 10^4^	NOG	4.65 × 10^3^	5.3 × 10^3^
Ola	S	8.15 × 10^5^	4.85 × 10^3^	7.4 × 10^3^	5.65 × 10^2^	NOG
Housing	T	4.75 × 10^5^	5.37 × 10^3^	4.4 × 10^3^	NOG	NOG
*p*‐value		<.001	<.001	<.001	<.001	<.001

Abbreviations: NOG, no observable growth, values with a and b subscripts in the same column represent maximum and minimum loads, respectively; TABC, Total aerobic bacteria count; TBC, total *Bacillus cereus* count; TEC, total *Escherichia Coli* count; TSC, total *Staphylococcus aureus* count; TSSC, total *Salmonella* count.

The study showed that the highest total aerobic bacteria count (TABC) was recorded at Zongo vending area (sample H) with a load of 1.85 × 10^7^ CFU/g whilst the Mawuli Estate vending area (sample P) had the least load of 2.75 × 10^4^ CFU/g. The study also showed that the highest *Staphylococcus aureus* count (TSC) was recorded at Zongo vending area with a load of 1.67 × 10^5^ CFU/g whilst the Mawuli Estate vending area (sample O) had the least load of 6.15 × 10^2^ CFU/g. In the case of the total *Escherichia coli* count (TEC), the highest contamination was recorded at the Poly vending area (sample L) with a load of 3.9 × 10^4^ CFU/g whilst the least load of 4.2 × 10^2^ CFU/g was recorded at Bankoe vending area (sample O). Mawuli Estate, Mirage, and Alaye vending areas had no observable TEC. For total *Bacillus cereus* count (TBC), Dave vending area (sample A) recorded the highest level of contamination with a load of 7.1 × 10^4^ CFU/g whilst the least load of 3.05 × 10^2^ CFU/g was recorded at Trafalgar (sample B). Dome, Market, SSNIT Flat, Mawuli Estate, and Housing vending areas had no observable TBC. For total *Salmonella* count (TSSC) also, Zongo vending area recorded the highest level of contamination with a load of 5.5 × 10^4^ CFU/g whilst the least load of 2.8 × 10^2^ CFU/g was recorded at Lokoe vending area (sample M). As shown in Table 1, eight vending areas had no observable TSSC.

The frequency of occurrence of bacterial species isolated from grilled beef sausage samples obtained from the various vending areas is presented in Table 3 below.

**TABLE 3:** Distribution and frequency of occurrence of bacterial species isolated from grilled beef sausage samples at different vending areas.

S/N	Isolated bacteria	Occurrence	Percentage (%)
1	*Staphylococcus aureus*	20	31.25
2	*Escherichia coli*	17	26.56
3	*Bacillus cereus*	15	23.44
4	*Salmonella*	12	18.75
	Total	64	100

A total number of 64 bacteria were isolated from the grilled beef samples. Four different bacteria genera were isolated. The bacteria isolated were *Escherichia coli*, *Staphylococcus aureus*, *Bacillus cereus*, and *Salmonella*. The table identifies the bacteria from the highest to the lowest frequency of occurrence. From the results obtained in Table 3, *Staphylococcus aureus* had the highest frequency of occurrence 20 (31.25%), followed by *Escherichia coli* with 17 (26.56%) and *Bacillus cereus* had 15 (23.44%). *Salmonella* had the lowest frequency of occurrence 12 (18.75%). Also, the morphological and biochemical (gram staining) characteristics of the organisms isolated from the grilled beef sausage and their preliminary identification are presented in Table 4 below.

**TABLE 4:** Morphological and biochemical characteristics of microorganisms isolated from grilled beef sausage.

Isolates	Color on media	Gram reaction	Arrangement
*Staphylococcus aureus*	Golden yellow on MSA	Gram‐positive cocci	Clustered
*Escherichia coli*	Pink on MCA	Gram‐negative rods	Separated
*Bacillus cereus*	Blue with cream centers on BSA	Gram‐positive rods	Separated
*Salmonella*	Cream with black dots on SSA	Gram‐negative rods	Clustered

Abbreviations: BSA, *Bacillus Cereus* selective agar; MCA, MacConkey; MSA, Manitol salt agar; SSA, *Salmonella Shigella* agar.

### 3.2 Fungal load in the grilled beef sausage

**FIGURE 2:**
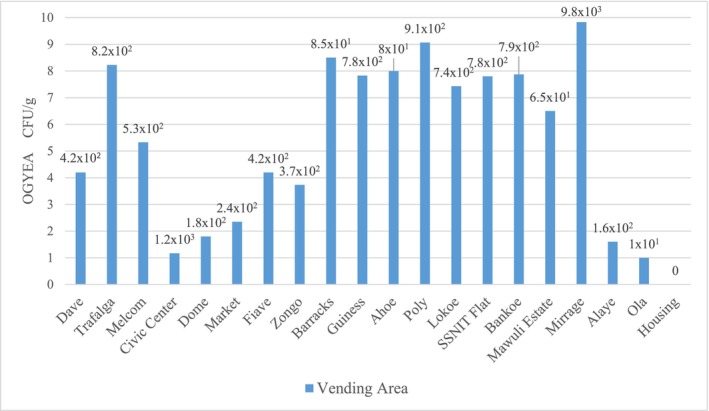
Mean fungal counts for sausage samples on OGYEA.

**FIGURE 3:**
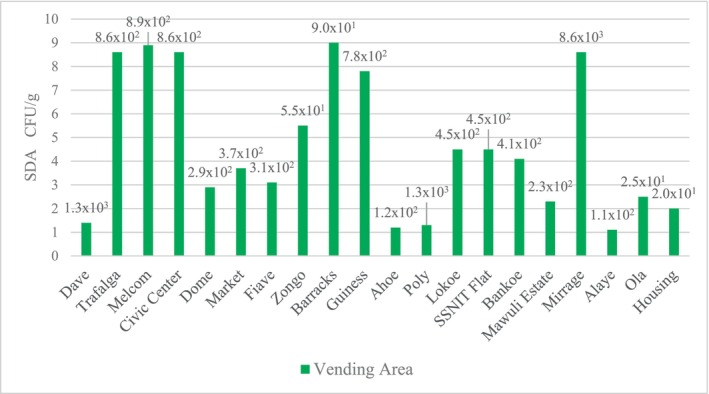
Mean fungal counts for sausage samples on SDA.

Figures 2 and 3 present the mean fungal counts for grilled beef sausages sampled from various vending areas. The results obtained indicated that, on oxytetracycline–glucose–yeast extract agar (OGYEA) (Figure 2), Mirage vending area (sample Q) recorded the highest total fungal counts of 9.83 × 10^3^ CFU/g whilst Housing vending area (sample T) recorded the least total fungal counts of 0.00 CFU/g.

Also, on Sabouraud dextrose agar (SDA) (Figure 3), the highest total fungal counts of 8.55 × 10^3^ CFU/g were recorded at Mirage vending area (sample Q) whilst the least total fungal counts of 2.0 × 10^1^ CFU/g were recorded at Housing vending area (sample T).

Furthermore, the identified fungi in the grilled beef sausage samples are presented in Table 5, where most of the samples had more than one fungal species.

**TABLE 5:** Isolated fungi from the grilled beef sausages.

Sample	*A. niger*	*A. Fumigatus*	*A. Ochraceus*	*A. Terreus*	*Fusarium* sp.	*Mucor* sp.	*Penicillium* sp.	*Rhodotorula* sp.	*Rhizopus* sp.
A	x						x		x
B	x				x	x	x		
C	x					x			x
D									x
E	x	x			x				x
F						x			x
G					x				
H	x				x	x		x	
I	x				x				x
J					x	x			x
K									
L	x					x	x		x
M	x					x			x
N					x	x			
O						x			x
P	x		x			x		x	
Q	x			x					x
R	x	x							
S	x			x					
T	x	x							
Total	13	3	1	2	7	10	3	2	11

*Note*: x = fungi presence. Sample A, Dave vending area; Sample B, Trafalgar vending area; Sample C, Melcom vending area; Sample D, Civic Center vending area; Sample E, Dome vending area; Sample F, Market vending area; Sample G, Fiave vending area; Sample H, Zongo vending area; Sample I, Barracks vending area; Sample J, Guiness; Sample K, Ahoe vending area; Sample L, Poly vending area; Sample M, Lokoe vending area; Sample N, SSNIT Flat vending area; Sample O, Bankoe vending area; Sample P, Mawuli Estate vending area; Sample Q, Mirage vending area; Sample R, Alaye vending area; Sample S, Ola vending area; Sample T, Housing vending area.

A total of nine fungal species (*Aspergillus niger*, *Aspergillus fumigatus*, *Aspergillus ochraceus*, *Aspergillus terreus*, *Fusarium*, *Mucor*, *Penicillium*, *Rhodotorula*, and *Rhizopus* species) belonging to six genera (*Aspergillus*, *Fusarium*, *Penicillium*, *Mucor*, *Rhodotorula*, and *Rhizopus*) were identified on both media.

**FIGURE 4:**
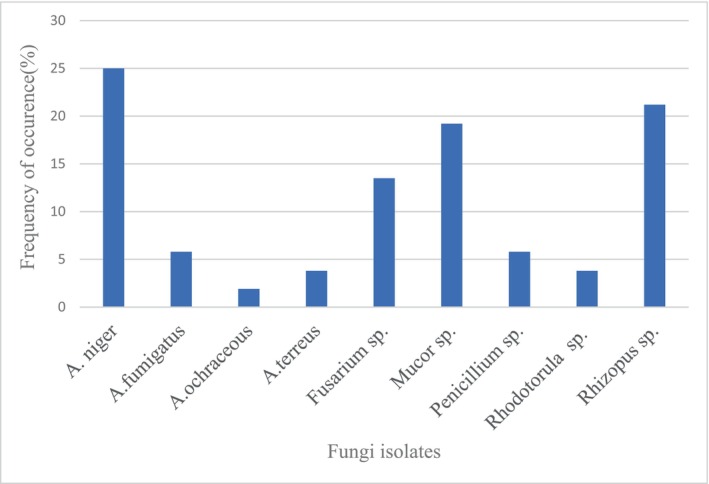
Frequency of occurrence (%) for Fungi isolates.

The percentage of occurrence of fungi isolated from the grilled beef sausage is presented in Figure 4.

**FIGURE 5:**
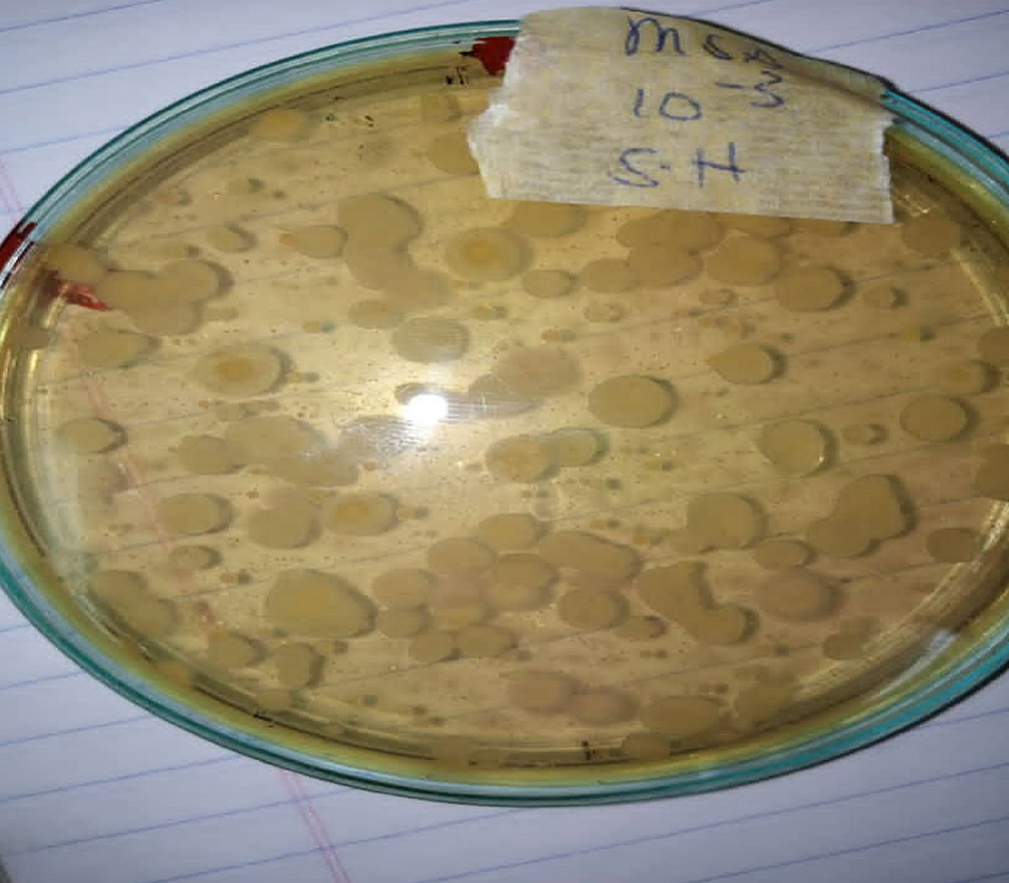
*Staphylococcus aureus* colonies on Mannitol salt agar.

**FIGURE 6:**
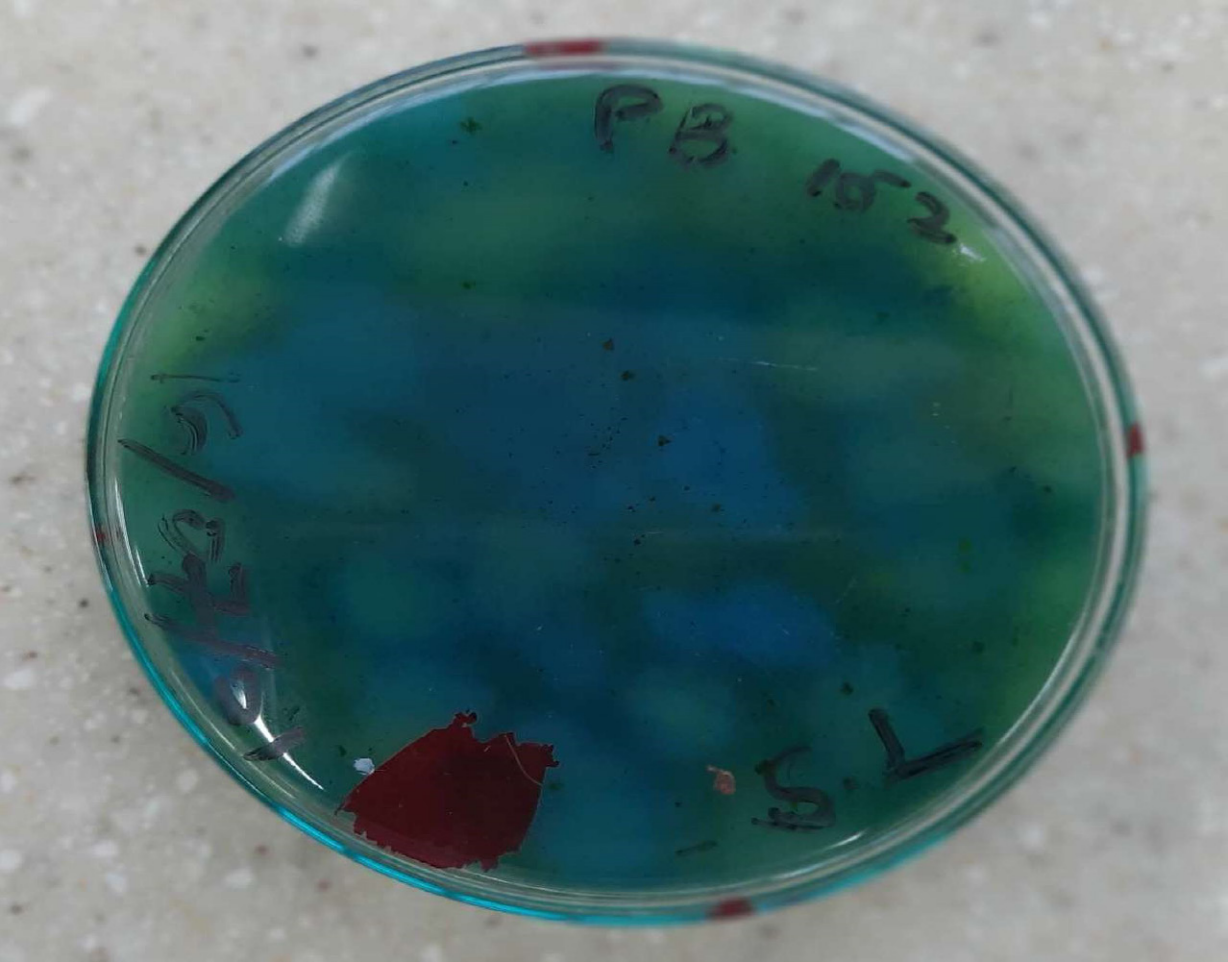
*Bacillus cereus* colonies on *Bacillus Cereus* selective agar.

**FIGURE 7:**
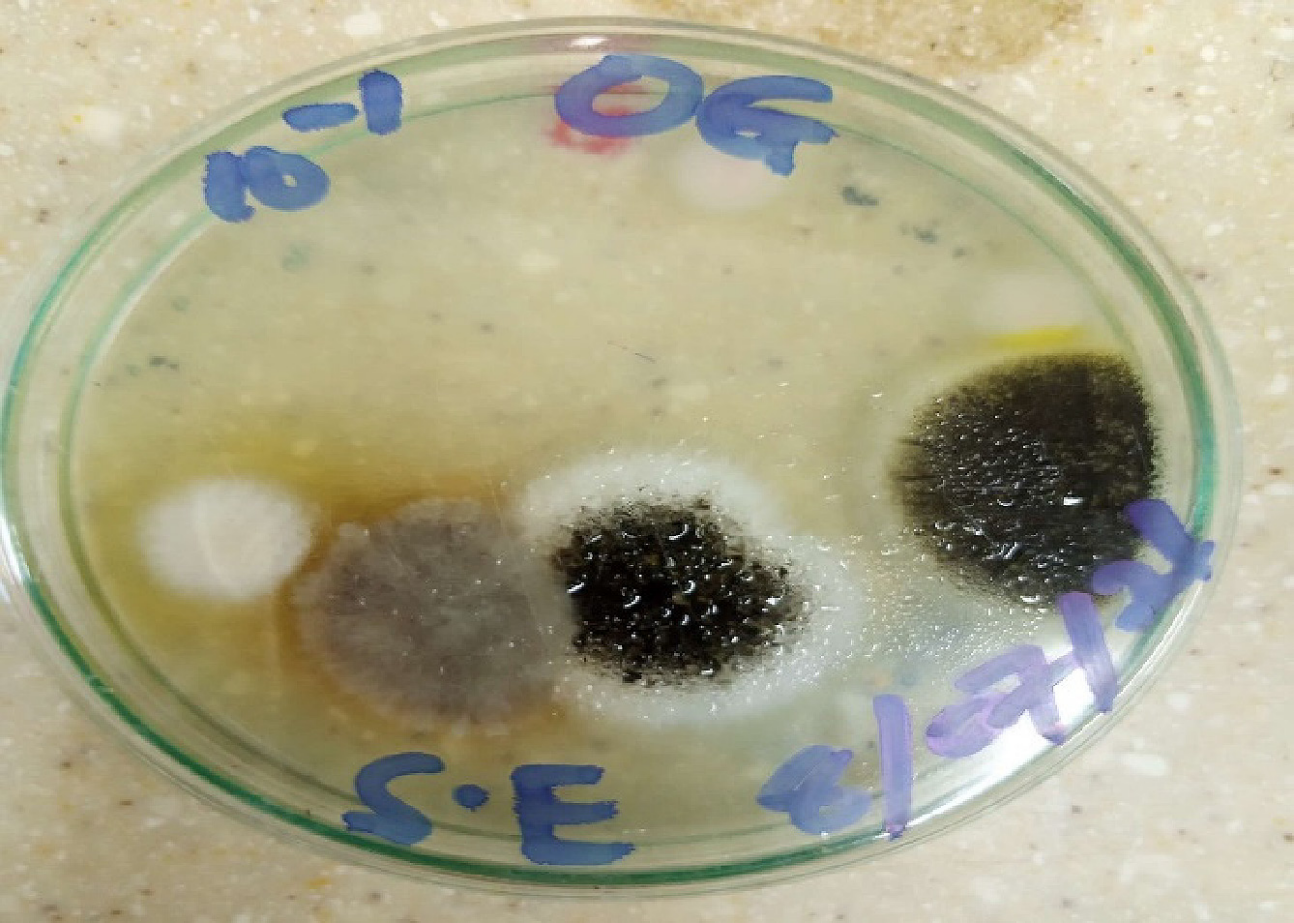
Macroscopic view of *Aspergillus* spp. and *Fusarium* spp.

**FIGURE 8:**
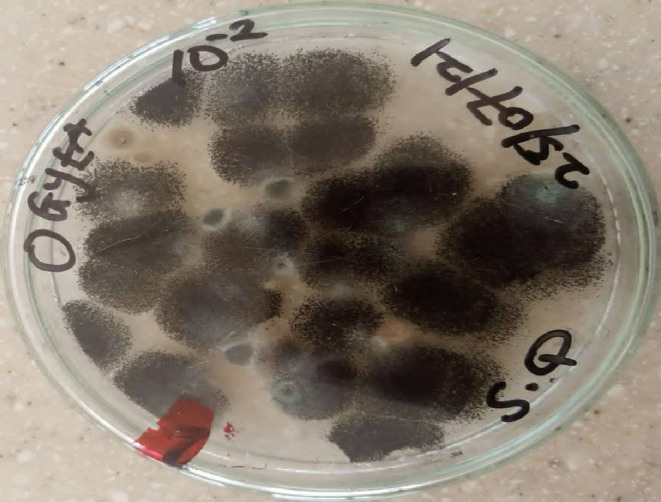
Macroscopic view of *Aspergillus niger*.

**FIGURE 9:**
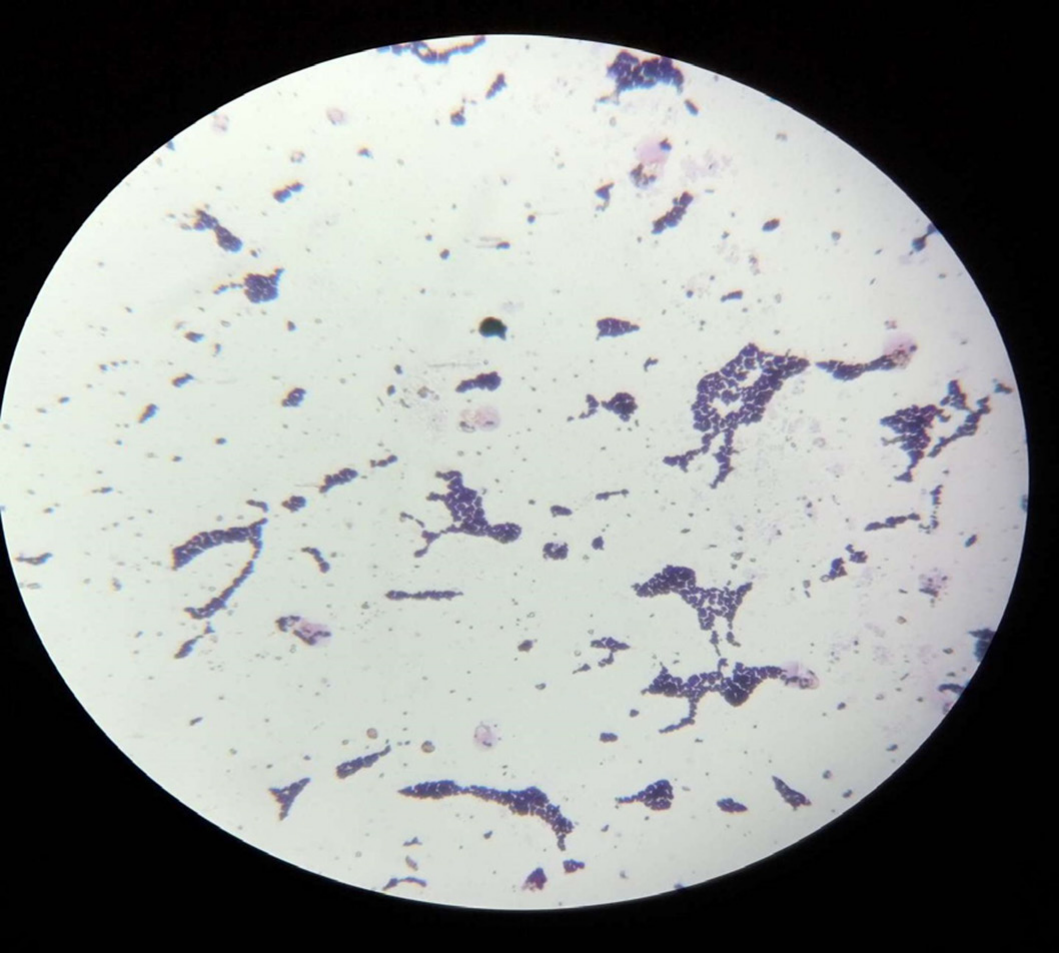
Microscopic view of *Staphylococcus aureus* (×1000).

**FIGURE 10:**
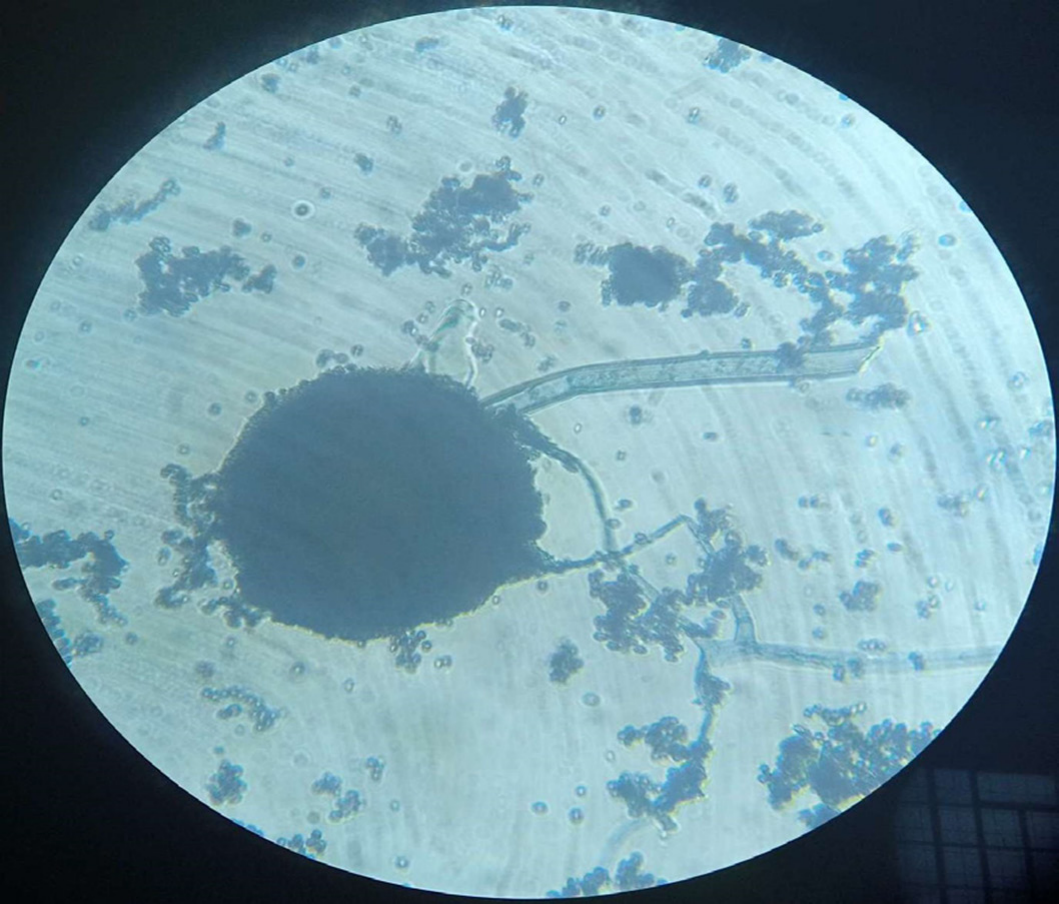
Microscopic view of *Aspergillus niger* (×400).

*Aspergillus niger* (25%) was the most frequently isolated fungi followed by *Rhizopus* sp. (21.2%), *Mucor* sp. (19.2%), *Fusarium* sp. (13.5%), *Aspergillus fumigatus* (5.8%), *Penicillium* sp. (5.8%), *Aspergillus terreus* (3.8%), *Rhodotorula* sp. (3.8%), and *Aspergillus ochraceus* (1.9%) was the least fungal isolate recorded in this survey. Below are also plates presented for *Staphylococcus aureus* (Figure 5), *Bacillus cereus* (Figure 6), *Aspergillus* spp., *Fusarium* spp. (Figure 7), *Aspergillus niger* (Figure 8), microscopic view of *Staphylococcus aureus* (Figure 9), and microscopic view of *Aspergillus niger* (Figure 10).

## 4 DISCUSSION

Microscopic organisms are incredibly diverse and constitute complex communities with numerous species of prokaryotes such as bacteria and archaea, unicellular eukaryotes such as fungi, protists, and algae, and some small multicellular eukaryotes (Mestre & Höfer, 2021), whose presence in food could become a biological hazard (Osimani et al., 2013). The results from the microbiological quality of street‐vended grilled beef sausages showed that total aerobic bacteria count (TABC) ranged from 2.75 × 10^4^ CFU/g to 1.85 × 10^7^ CFU/g for all samples collected. The analysis of variance revealed that there were statistically significant differences (*p* < .05) among the total viable bacteria count of grilled beef sausages sampled from the different vending areas. The Ghana Standard Authority (2019) specifies that the total aerobic bacteria count should be less than or equal to 10^4^ CFU/g for ready‐to‐eat foods. The results obtained indicated that 15 samples representing 75% were above the acceptable microbiological limits whilst only five samples representing 25% were within the acceptable limits.

The microbial quality of meat products is influenced by several factors. The higher microbial loads in most of the samples could be attributed to unsanitary practices and inadequate grilling by the kebab vendors. The total aerobic bacteria count represents the total number of viable pathogenic microorganisms (bacteria, yeasts, and molds) present in a sample and it is an important aspect in determining the hygienic environment during the storage and processing of beef sausages. Upon observation, the beef sausages grilled by the kebab vendors were left uncovered or not stored in glass showcases, exposing them to dust and flies, which are carriers of microorganisms, possibly contributing to the high microbial load. As suggested by Ayamah et al. (2021), hygienic procedures such as covering food from flies and dust, cleaning fingernails, and using aprons and head cups by street food vendors are one of the key measures that help to prevent foodborne contamination.

Additionally, as meat typically contains the nutrients needed for both microbial growth and their metabolism, it is more likely to be contaminated by microbes (Ayamah et al., 2021). Similar research carried out in Kalyobia Governorate by Shaltot et al. (2015) showed a mean value of 1.33 × 10^6^ CFU/g for aerobic plate count (APC) in 20 street‐vended sausages. Also, this study is in line with reports by Chingoma et al. (2016), Falegan et al. (2017), EI‐Hassan et al. (2018), and Alabi et al. (2021) in which the total aerobic bacteria count for ready‐to‐eat meat products ranged from 1.7 × 10^4^ CFU/g to 1.39 × 10^5^ CFU/g, 9.8 × 10^4^ CFUL/ml to 2.85 × 10^5^ CFU/mL, 1.4 × 10^4^ CFU/mL to 2.95 × 10^5^ CFU/ml, and 1.5 × 10^6^ CFU/g to 7.8 × 10^6^ CFU/g, respectively. In contrast to the findings of this study, Aseigbu et al. (2015) reported lower total aerobic counts in the range 0.9 × 10^2^–0.2 × 10^4^ CFU/g from barbecued beef sausage in Johannesburg, South Africa.

The results on the enumeration of total *Staphylococcus aureus* counts (TSC) for all the samples of street‐vended grilled beef sausages ranged from 6.15 × 10^2^ CFU/g to 1.67 × 10^5^ CFU/g. Statistically, there were significant differences (*p* < .05) among the TSC of grilled beef sausages sampled from different vending areas. Ayamah et al. (2021) also reported a similar high *S. aureus* load of 3.10 × 10^2^ to 2.96 × 10^7^ CFU/g with no significant difference among the results in the KNUST campus and its environs in Ghana. According to Ghana Standard Authority (2019), the limit for *Staphylococcus aureus* is satisfactory for samples <20 CFU/g, and the acceptable upper limit for samples with 20 to ≤10^3^ CFU/g and >10^3^ CFU/g is considered unsatisfactory for ready‐to‐eat foods. The results obtained from the microbial assay indicated that 55% of the sampled grilled beef sausages were within the borderlines which indicates the maximum acceptable limits. The results also revealed that 45% of the sampled grilled beef sausages were above the acceptable limit (unsatisfactory). This indicates that all the samples analyzed in this study were contaminated with *S. aureus*. This is almost similar to the results obtained by Atter et al. (2020) from Ghana, who reported that out of 25 ready‐to‐eat grilled beef sausage samples purchased from different vendors in the Greater Accra Region, 22 of them were contaminated with *S. aureus*. Also, the current results conform with the results obtained by Shaltot et al. (2015), Falegan et al. (2017), EI‐Hassan et al. (2018), Chingoma et al. (2016), Karoki et al. (2018), Madoroba et al. (2021), Ananias and Roland (2017), and Unachukwu et al. (2019) who also recorded *Staphylococcus aureus* as the most frequently isolated bacteria and highest percentage occurrence in ready‐to‐eat meat products. However, these results disagree with the findings of Alabi et al. (2021) who recorded the lowest percentage of *Staphylococcus aureus* (15.8%) in ready‐to‐eat roasted beef in Ibadan, Nigeria.

The presence of *Staphylococcus aureus* in the grilled beef sausage samples suggested that the street kebab vendors were not practicing good personal hygiene. In most studies, *Staphylococcus aureus* is one of the major known pathogens identified on hands (Rabbi et al., 2011; Shojaei et al., 2006). It has also been proven that majority of street‐vended food handlers are agents of *S. aureus* (Rabbi et al., 2011). It was observed that most of the kebab vendors handled the sausages with their bare hands while cutting them, and the use of old newspapers for wrapping the grilled beef sausages could be a contributing factor. Also, the presence of *Staphylococcus aureus* could be attributed to the raw ingredients used (such as raw sliced onion). Furthermore, street kebab vendors hardly wash their hands, especially when there are a lot of customers waiting to be served. According to Luu and Michiels (2021), it appears that foods that are extensively handled manually are more likely to be contaminated with *S. aureus*, bacteria that lives naturally on human skin, in the nose, and ears. The results on the enumeration of total *Escherichia coli* (TEC) for all the samples of street‐vended grilled beef sausages ranged from 4.2 × 10^2^ CFU/g to 3.9 × 10^4^ CFU/g. There were statistically significant differences (*p* < .05) among the TEC of grilled beef sausages sampled from the different vending areas. According to Ghana Standard Authority (2019), the limit of total *Escherichia coli* of <20 CFU/g is satisfactory, 20 to ≤10^2^ CFU/g is within the acceptable upper limit, and >10^2^ CFU/g is considered unsatisfactory for ready‐to‐eat foods. The results obtained from the microbial examination indicated that 75% of the sampled grilled beef sausages were above the acceptable limits, and 25% were within the acceptable limits.

The presence of a high *Escherichia coli* count in kebab samples indicates fecal contamination and poor hygienic practices by food vendors (Ayamah et al., 2021). In this study, the frequency of occurrence of *Escherichia coli* was 26.56%. This finding was almost similar to that obtained by Onuorah et al. (2015) who reported a 34.3% occurrence of *Escherichia coli* in tsire‐suya (grilled beef) in Nigeria. However, this finding was in disagreement with that of Karoki et al. (2018) and Falegan et al. (2017) who reported *Escherichia coli* as the least level of occurrence in ready‐to‐eat meat products (1.6% and 5%, respectively). The presence of *E. coli* in most of the grilled beef sausages analyzed suggested that the kebab vendors did not follow proper personal hygiene practices. According to Ayamah et al. (2021), vendors rarely follow hygienic practices which might have contributed to the high level of *E. coli* count in the Kebab samples. In humans, some strains of *E. coli* when consumed through food could result in gastroenteritis and diarrhea (Kortei et al., 2020). Spices such as pepper which are added to the grilled beef sausage during serving may also be another source of contamination because according to Falegan et al. (2017), spices may even serve as a source.

Furthermore, other prominent sources of contamination include insufficient cooking, and the use of contaminated utensils, equipment, and raw ingredients (Kothe et al., 2016). *E. coli* is an indicator organism used to determine the sanitary quality of foods. According to Chingoma et al. (2016), the presence of *E. coli* compromises the microbiological quality of the sausages.

The total *Bacillus cereus* counts (TBC) ranged from 3.05 × 10^2^ CFU/g to 7.1 × 10^4^ CFU/g. Statistically, there were significant differences (*p* < .05) among the TBC of grilled beef sausages sampled from different vending areas. In Ghana, GSA (2019) specifies <10^2^ CFU/g as satisfactory and 10^2^ to ≤10^4^ CFU/g as within the acceptable upper limits (borderline), and > 10^4^ CFU/g as unsatisfactory for total *Bacillus cereus* in ready‐to‐eat foods. The results obtained from the microbial examination of the samples analyzed revealed that 10% and 90% were above and within the acceptable limits, respectively, per the guidelines of the Ghana Standard Authority (2019). The presence of *Bacillus cereus* contamination might be due to inadequate grilling of beef sausages. According to Atter et al. (2020), these kebab vendors probably believe that the sausage is cooked during its production process and may, thus, be consumed fresh, so it does not require a lot of grilling to get rid of potential pathogens. Due to their high heat resistance, *B. cereus* spores are not destroyed by cooking at the right temperatures, even though doing so would kill most foodborne pathogens, including their vegetative cells (Dubey et al., 2021). The frequency of occurrence of *Bacillus cereus* in this study was 23.44%. These findings are related to the work by Karoki et al. (2018) who reported a relatively lower percentage of 19.5% of *Bacillus cereus* contamination in African sausages in Kenya. However, these findings contradict that of Falegan et al. (2017) and Alabi et al. (2021) who recorded the lowest number of *Bacillus* spp. occurrences with 10% and 5.3%, respectively, in suya (roasted beef).

The total *Salmonella* count (TSSC) ranged from 2.8 × 10^2^ CFU/g to 5.5 × 10^4^ CFU/g. Statistically, there were significant differences (*p* < .05) among the TSSC of grilled beef sausages sampled from the different vending areas. Ghana Standard Authority (2019) emphasized that *Salmonella* spp. should not be detected in 25 g of ready‐to‐eat foods which is considered to be satisfactory for human consumption otherwise it would be considered unsatisfactory. The results obtained in this study showed that 12 samples representing 60% were contaminated with *Salmonella* and therefore unsatisfactory for human consumption. However, Chingoma et al. (2016) from Zimbabwe reported a relatively lower percentage of 38.9% contamination with *Salmonella* was detected in samples of street‐vended sausages. The presence of *Salmonella* in grilled beef sausages could be attributed to inadequate grilling, poor handling practices, and cross‐contamination. Most kebab sellers, based on observation, do not only sell grilled beef sausages to customers but also grilled meat. As a result, they use the same knife to cut the grilled beef sausage and meat, as well as the fresh meat, potentially resulting in cross‐contamination. As suggested by Falegan et al. (2017), the majority of persons involved in the processing and sale of ready‐to‐eat meat products are usually illiterates without formal training in food preparation, which is necessary for the hygienic handling of foods. *Salmonella* is found in the gut and has been linked to acute bacterial diarrhea and food poisoning and is not acceptable in meat and meat products (Ikechukwu et al., 2019). According to Ghana Standards Authority, *Salmonella* should not be detected in ready‐to‐eat foods; therefore, grilled beef sausages contaminated with *Salmonella* spp., in this study, were microbiologically unsafe for human consumption. This is because some *Salmonella* serotypes have a low infectious dose which means they can still cause disease even without proliferating in the food (Luu & Michiels, 2021).

The presence of yeasts and molds usually contaminate foods and subsequently produce mycotoxins which are most often not eliminated through processing. The results obtained for the mean fungal counts on OGYEA ranged from 0.0 CFU/g to 9.83 × 10^3^ CFU/g. Statistical analysis of the mean fungal counts on OGYEA showed no significant difference (*p* = .28) among the various vending areas. A similar trend was also observed on SDA, the mean fungal counts ranged from 2.0 × 10^1^ CFU/g to 8.55 × 10^3^ CFU/g. Statistical analysis of the mean fungal counts on SDA also showed no significant difference (*p* = .33) among the various vending areas. Fungal counts recorded in this work were in the same range of values of 1.0 × 10^3^ to 8 × 10^3^ CFU/mL reported by EI‐Hassan et al. (2018), as counts of processed meat products (Tsire) in Nigeria. Despite the fact that all of the samples analyzed were within the acceptable range of microbiological counts for ready‐to‐eat foods (<10^4^ CFU/g) as prescribed by the International Commission for Microbiological Specification of Foods (ICMSF, 1998), the presence of fungal species in the grilled beef sausages constitutes a public health risk for consumers. The highest fungal counts were recorded at the Mirage vending area, this could be attributed to the sausages being exposed to dust and flies, as well as the kebab vendor's poor personal hygiene. According to Osakue et al. (2016), the presence of fungal species in ready‐to‐eat food indicates a deplorable state of poor hygiene and sanitary practices used in the processing and sales of these food products. Therefore, the presence of fungal species in the grilled beef sausages implies poor hygienic practices of food handlers and insufficient grilling.

Mold and yeast attacks on food are more severe since they may develop easily not only at room temperature but also in cool, chilled storage settings (Petruzzi et al., 2017). Even though molds are involved in the spoilage of many kinds of foods, according to Minj et al. (2020), yeasts play a significant role in the production of alcoholic beverages, due to the capacity to gather high levels of ethanol and the production of aromatic compounds. As stated by Noumavo et al. (2022), the presence of molds and yeasts in food should not be disregarded because many of them, in particular mycotoxinogenic molds (aflatoxins, ochratoxins, and fumonisins production), can be harmful to human health when consumed in large quantities. From the results obtained in this study, *Aspergillus niger* had the highest frequency of occurrence (25%). This is congruent with the results obtained by Makhlouf et al. (2019) who claimed that *Aspergillus* was the dominant genus in samples of spices used in meat processing that were analyzed. Also, EI‐Hassan et al. (2018) recorded *Aspergillus niger* as the most frequent occurrence of species in processed meat products (Tsire) sold in Nigeria. Oranusi and Braide (2012) stated that the mishandling of food products could lead to the proliferation of fungi beyond acceptable limits. However, Algammal et al. (2021) found *A. flavus* as the most predominant mold species isolated from the processed meat samples. Furthermore, the presence of fungi such as *Aspergillus niger* in the food indicates danger to public health because many of these fungi are toxin‐producing organisms (Osakue et al., 2016).

Even though low counts of species of *Penicillium* were isolated, it is still of pathological importance. *Penicillium* is in the Deuteromycetes made up of diverse fungal genera of the ascomycetous fungi and contains more than 350 species. More than 80 *Penicillium* species are documented toxin producers (Agriopoulou et al., 2020); the most important are ochratoxin A, citreoviridin, penitrem A, roquefortine, and secalonic acids. The genus has major importance in the natural environment as well as the food and drug production industry. The preponderant fungal species isolated in this study with toxigenic potential, human health, and pathological importance were *A. niger*, *A. fumigatus*, *A. ochraceus*, *Fusarium verticillioides*, *F. oxysporum*, and *Penicillium* spp. Three major genera of fungi are identified to produce mycotoxins, including *Aspergillus*, *Fusarium*, and *Penicillium*, although other genera also produce toxic compounds (Ahmed et al., 2013). *Aspergilli* and *Penicilliums* are linked to agricultural commodities during postharvest storage (Agriopoulou et al., 2020; Balendres et al., 2019). Zearalenone causes human uterotrophic (antireproduction) effects in animals and pigs (Agriopoulou et al., 2020).

*Fusarium* species isolated from beef sausages produce several mycotoxins such as biologically active trichothecenes which when ingested in high concentrations, cause vomiting and diarrhea in humans. Trichothecenes are also associated with reduced weight gain and immune dysfunction in animals (Huang et al., 2019). Another *Fusarium* species *F. verticillioides* (*F. moniliforme*) isolated from beef sausages in the study produces fumonisins which have neurotoxic effects in animals and are associated with esophageal cancer (Schrenk et al., 2020).

## 5 CONCLUSIONS

This exploratory survey demonstrated that all the vending areas of grilled beef sausages serve as potential vehicles of foodborne pathogens that present risks of foodborne illness to humans such as *Salmonella* spp., *Staphylococcus aureus*, *Bacillus cereus*, and *Escherichia coli* and also the fungal species such as *Aspergillus* spp. and *Rhizopus* spp. The survey also demonstrates that the materials such as used newspapers, the use of bare hands, and exposure to the environment constitute contamination of grilled beef sausages. For this reason, training regimes should be organized for the vendors of this product with regular inspection and appropriate supervision to ensure consumer safety in the consumption of the product. Also, periodic follow‐up visits to inspect hygienic vending areas should be carried out to ensure that the vendors adhere to their training.

## AUTHOR CONTRIBUTIONS

**Banabas Nkekesi:** Data curation (equal); investigation (equal); methodology (equal); resources (equal); writing – original draft (lead); writing – review and editing (lead). **Priscilla Amenya:** Conceptualization (equal); data curation (equal); resources (equal); supervision (equal); writing – original draft (equal). **George Aboagye:** Conceptualization (lead); data curation (lead); investigation (lead); methodology (lead); resources (equal); supervision (equal); validation (equal); writing – review and editing (equal). **Nii Korley Kortei:** Data curation (equal); investigation (equal); methodology (equal); resources (equal); validation (equal).

## FUNDING INFORMATION

The authors declare that no funding or grant was obtained for the study.

## CONFLICT OF INTEREST STATEMENT

The authors declare no competing interest from conception to the finalization of the study.

## ETHICAL APPROVAL AND CONSENT TO PARTICIPATE

This study did not involve any human or animal testing. Ethical review was sought from the University of Health and Allied Sciences, Research Ethics Committee (UHAS‐REC) of the Institute of Health Research. Following approval of the research, the institute issued the certificate number UHAS‐REC A.9 [51] 20–21. The grilled beef sausage samples were purchased from vendors by convenience sampling, mimicking similar instances of patronage from the general public, whilst handling and hygienic practices were also observed.

## CONSENT FOR PUBLICATION

The manuscript does not contain any individual's data; therefore, consent for publication is not applicable.

## Data Availability

Additional information or data related to the study shall be made available on request.
